# High-Dose-Rate Brachytherapy for Primary Treatment of Refractory Proliferative Verrucous Leukoplakia of the Hard Palate

**DOI:** 10.7759/cureus.15696

**Published:** 2021-06-16

**Authors:** Jahan J Mohiuddin, Rabie M Shanti, Faizan Alawi, Brian M Chang, Jaclyn Marcel, Neil K Taunk, John N Lukens

**Affiliations:** 1 Radiation Oncology, University of Pennsylvania, Philadelphia, USA; 2 Otolaryngology - Head and Neck Surgery, University of Pennsylvania, Philadelphia, USA; 3 Dermatopathology, Division of Oral and Maxillofacial Pathology, University of Pennsylvania, Philadelphia, USA; 4 Oral and Maxillofacial Surgery, University of Pennsylvania, Philadelphia, USA

**Keywords:** oral leukoplakia, brachytherapy, radiotherapy, proliferative verrucous leukoplakia, oral cavity cancer

## Abstract

Oral proliferative verrucous leukoplakia (PVL) is a rare, progressive form of leukoplakia with a high rate of malignant transformation. No therapies are known to lower the rate of malignant transformation and prevent a recurrence.

An 84-year-old patient with a years-long history of symptomatic PVL of the hard palate refractory to CO2 laser ablation presented to the radiation oncology clinic for consideration of non-surgical management. High dose rate brachytherapy was used to deliver 36 Gy in 12 fractions to the hard palate using an Ir-192 source with a custom-molded applicator.

By three months of follow-up, the patient had complete regression of the PVL and resolution of acute mucositis. With 18 months of follow-up, the patient remains disease- and symptom-free without toxicities of treatment.

High dose rate surface applicator brachytherapy is a feasible and potentially effective treatment for oral PVL, yielding durable control with low long-term toxicity.

## Introduction

Oral proliferative verrucous leukoplakia (PVL) is a rare, aggressive type of oral leukoplakia that is progressive, multifocal, and known to have a high risk of malignant transformation. Our group sought a novel treatment paradigm for a symptomatic and high-risk lesion of the hard palate diagnosed as PVL within the premalignant phase of its progression. In this case report, initially presented at the 2019 Annual Meeting of the American Brachytherapy Society on June 8, 2018 (Miami, Fl), we describe the treatment of refractory PVL with high-dose-rate surface-applicator brachytherapy.

## Case presentation

**Figure 1:**
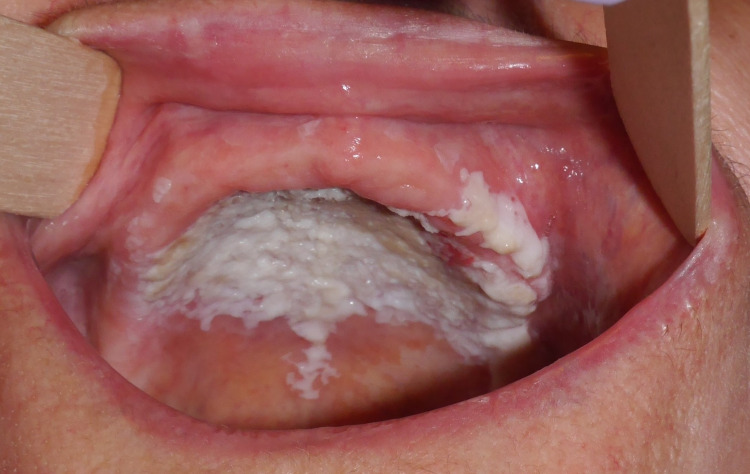
Thick, homogeneous, non-ulcerated, verrucous leukoplakia coats the hard palate and extends to the bilateral maxillary gingiva.

**Figure 2:**
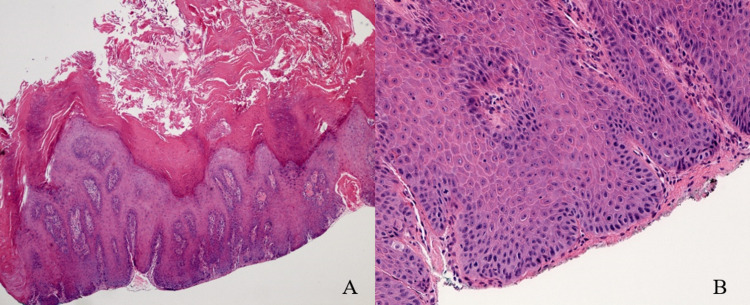
Hematoxylin and eosin stained microscopy at (A) x40 magnification showing markedly hyperkeratotic, acanthotic and hyperplastic stratified squamous epithelium, with elongated rete pegs, and (B) x200 magnification showing mild dysplastic changes including nuclear hyperchromasia and pleomorphism and mitoses within the lower one third of the epithelium.

An 84-year-old patient initially presented to the oral and maxillofacial surgery clinic in 2016 with a years-long history of PVL surveyed with biopsies. The patient had thick verrucous leukoplakia involving the hard palate and bilateral maxillary gingiva (Figure 1), initially diagnosed microscopically as high-grade dysplasia (Figures 2A, 2B). The lesion was refractory to multiple courses of resection using CO2 laser evaporation, and was highly vascular-often bleeding during the ablative procedure. Immediately following ablation, the patient returned to pre-treatment disease-related symptoms including discomfort while eating, and could not comfortably wear a maxillary denture due to the thickness of the lesion. Surgical resection would have resulted in an extensive defect with denuded bone, requiring flap-based reconstruction. There is little medical evidence of the benefit of such an aggressive surgical intervention. Therefore, she was referred to the radiation oncology clinic to be considered for non-surgical management.

Given that the PVL was limited to the hard palate and maxillary gingiva, high-dose-rate (HDR) brachytherapy was planned to treat the local disease with sparing of the uninvolved oral mucosa. HDR brachytherapy is a form of temporary radiation in or near a target, with treatment performed often in minutes per day.

**Figure 3:**
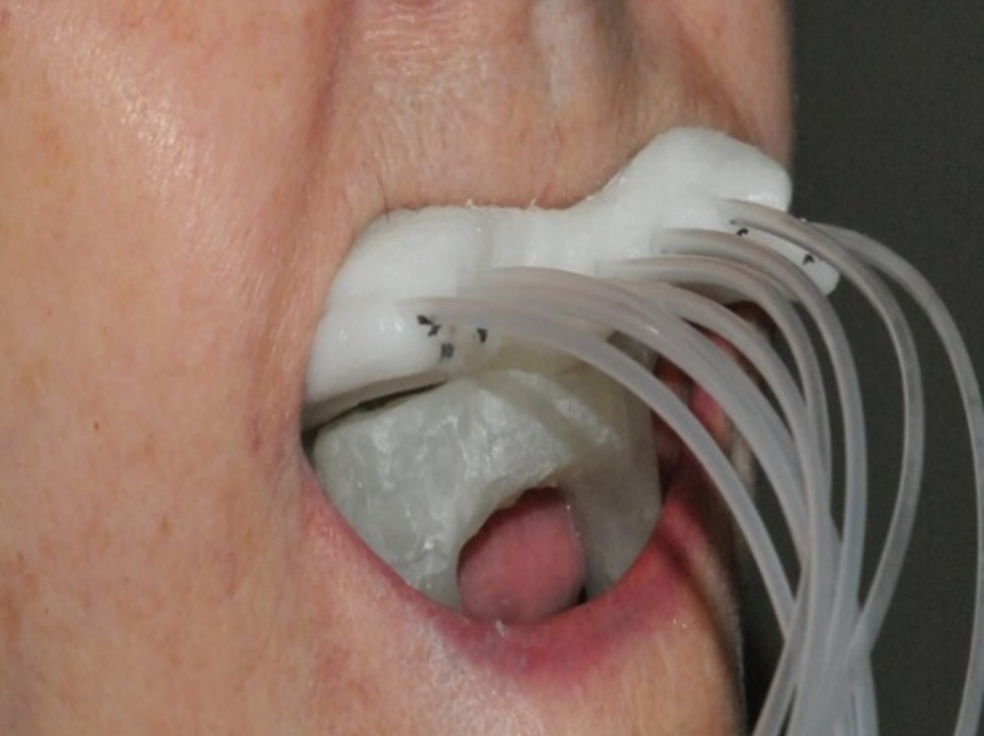
Brachytherapy surface applicator (superior, with catheters emerging from it) and oral appliance (inferior, used to depress tongue and separate away lower oral cavity) in place.

**Figure 4:**
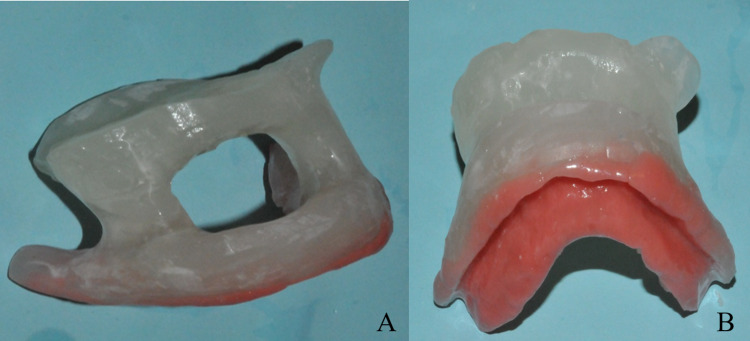
An (A) anterior view, and (B) inferior view of a custom appliance to displace non-target structures from the radiation field. The appliance displaces the tongue, mandible, and lower lip from the Ir-192 source and also apposes the applicator against the hard palate.

A surface mold applicator with seven embedded HDR catheters was created from the Aquaplast RT^TM^ thermoplastic mold (QFix, Avondale, PA) (Figure 3). A maxillofacial prosthodontics specialist fabricated a custom positioning stent that displaced the tongue, lower lip, and mandible, and also apposed the HDR surface applicator to the mucosa of the palate (Figure 4). To craft the positioning stent, working casts were created from maxillary and mandibular impressions. The stent was fabricated with a visible-light-cured resin (Triad®; Dentsply Sirona, USA). Vinylpolysiloxane Soft Relining Material (GC America, USA) was incorporated to ensure patient comfort and stability of the stent. No shielding material was incorporated into the stent. A hole in the anterior segment allowed the patient to protrude the tongue during treatment. 

**Figure 5:**
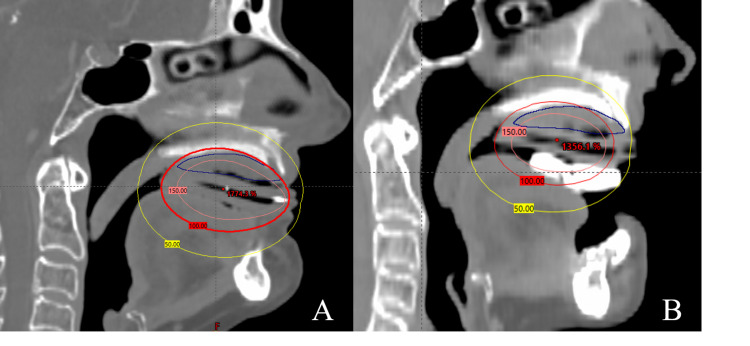
Isodose lines for treatment plan (A) without oral appliance, and (B) with oral appliance in the sagittal view. The target volume (CTV) is in blue. The 100% isodose of radiation (red line) covers the entire target. The oral appliance significantly reduces high-dose and low-dose scatter to the rest of the oral cavity.

CT-based planning was used to create a clinical target volume (CTV) encompassing the disease and mucosa of the hard palate and bilateral maxillary gingiva, while sparing the remainder of the oral cavity (Figure 5). Electron scatter from the positioning stent was not a concern for two reasons: (1) the Hounsfield Units of the resin in the positioning stent are equivalent to that of bone, and (2) 5 mm of Aquaplast separated the superior surface of the positioning stent from the mucosa of the hard palate. The patient was prescribed 48 Gy in 16 fractions to the CTV using an Ir-192 source with treatment once per day. She was treated daily for the first seven fractions, but due to acute mucositis, she was switched to an every-other-day treatment regimen. Due to the development of grade 3 mucositis requiring tramadol and viscous lidocaine, the treatment was stopped early at 36 Gy in 12 fractions. 

**Figure 6:**
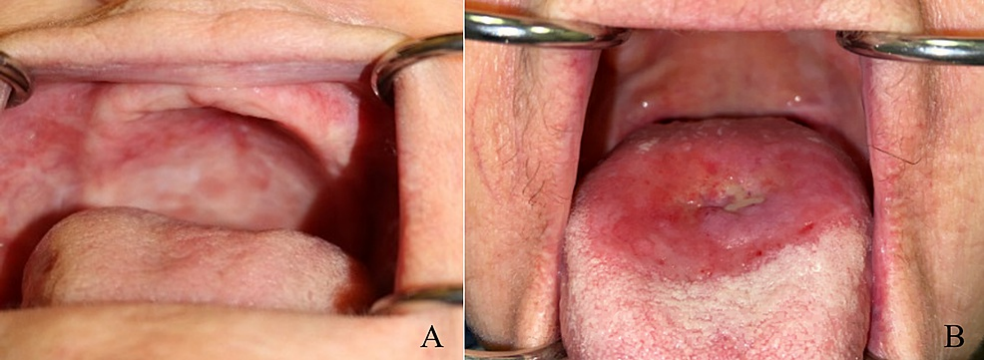
Post-treatment photographs at (A) three months of follow-up showing complete resolution of the target lesion, and (B) nearly 36 months of follow-up showing a late ulcer and depapillation of the posterior tongue. The clear line of demarcation roughly correlates with the 125%-133% isodose line.

After three-months of follow-up, the patient had a complete response to the treatment and had fully recovered from the acute toxicities of radiotherapy (Figure 6A). After 18 months of follow up, the patient remained free of recurrence with substantially improved quality of life. By 24 months of follow up, the patient developed a small ulcer on the posterior dorsal tongue. After nearly 36 months of follow up, an area of depapillation had developed surrounding the ulcer, which was now painful (Figure 6B). The late ulceration is being managed conservatively with dexamethasone oral solution and viscous lidocaine.

## Discussion

Oral PVL is a rare, aggressive type of oral leukoplakia that is progressive, multifocal, and known to have a high risk of malignant transformation [1-5]. The first mention of this disease in the literature is a 30-patient case series published in 1985 by Hansen et al. describing the clinical course of PVL [1]. PVL was characterized as “slow-growing, persistent, and irreversible…recurrence is the rule.” The authors reported that PVL exists as a spectrum between hyperkeratosis and invasive squamous cell carcinoma.

The etiology of PVL is unclear, and it does not appear to share the same risk factors as oral leukoplakia, such as tobacco, alcohol, or betel nut use [2]. In a 148-patient retrospective study, there was no statistically significant difference in the HPV positivity rate between PVL (24.1%) and oral leukoplakia (25.5%), suggesting that HPV infection is not a driving factor for the development of PVL [2]. A systematic review and meta-analysis of the literature reported that the cumulative malignant transformation rate of PVL is 49.5% with an annual rate of 9.3% per year, which was the highest among the potentially malignant disorders of the oral cavity [3]. Aggressive interventions do not appear to lower the malignant transformation rate [4]. Borgna and colleagues reported that conservative management in the premalignant phase of PVL resulted in a similar rate of malignant transformation compared to surgical excision [5].

In the first report of PVL in the literature by Hansen et al, at least 18 out of 30 patients received radiation, sometimes as monotherapy and others combined with surgery [1]. Little detail was given about the radiation used, but at least one patient received radium treatment with interstitial needles, an obsolete form of brachytherapy. Interestingly, the only patient with long-term control of the disease was treated with radium needles to the buccal mucosa, though the authors note it is possible that the patient did not actually have PVL.

Beyond the scant detail in the Hansen study, we are not aware of reports of the use of brachytherapy as a definitive treatment for PVL in the pre-malignant phase. Interstitial brachytherapy for oral cavity carcinomas is well-described, where it has been used as both a definitive and boost modality [6-8]. In contrast, surface applicator brachytherapy for hard palate cancers is much less prevalent in the literature. Unetsubo et al. reported the outcomes of two patients who received 30 Gy of external beam radiotherapy followed by a surface mold brachytherapy boost for hard palate cancer [9]. One of the two patients developed late grade 3 ulceration of the palatal mucosa while toxicity was unreported for the second patient. Van Gestel et al. reported a case of an elderly man with carcinoma of the upper alveolar ridge spreading to the hard palate [10]. He was treated with surface-applicator HDR brachytherapy to 48 Gy in 12 fractions and developed high-grade acute mucositis after eight fractions. After five months of follow-up, the patient had a complete remission of the tumor but had not fully resolved the mucositis. Wong et al. treated a patient with hard palate cancer using surface brachytherapy to 40 Gy in eight fractions, with three fractions per week [11]. The patient developed expected mucositis, which resolved, and had a complete response of the tumor with no clinically significant late toxicities. She did develop asymptomatic exposed bone over a torus palatinus adjacent to the tumor. While both the van Gestel and Wong case reports describe using a mold applicator, neither group created a second custom oral positioning stent as we did. As seen in Figure 5B, the positioning stent effectively displaces both the tongue and mandible, while simultaneously apposing the applicator firmly against the hard palate. This serves to substantially reduce incidental dose to the oral tongue, lower alveolar ridge, and mandible. 

Our patient had a confluence of factors making her disease amenable to treatment with high-dose-rate brachytherapy. She had a symptomatic disease that was not amenable to surgical intervention, thus warranting non-surgical management. Her disease was relatively confined to the surface of the hard palate, which allowed a uniform dose distribution to adequately cover the PVL--ideal for brachytherapy. Multifocal disease would have required external beam radiation which would cause more acute and potentially late toxicity, such as xerostomia.

There were several factors in choosing the initial dose and fractionation of 48 Gy in 16 fractions. Doses of 3-4 Gy per fraction are typically utilized when treating with brachytherapy to the oral cavity [12]. In this case, 3 Gy per fraction was selected given the sensitivity of the oral mucosa and palate in particular. A standard HDR brachytherapy dose for squamous cell carcinoma is 3 Gy x 17 fractions, and we scaled back by one fraction given that we were treating presumed carcinoma in situ [13]. Assuming an alpha/beta ratio of three for the oral cavity mucosa, 48 Gy in 16 fractions is equivalent to 57.6 Gy in 2 Gy fractions (EQD2; equivalent dose in 2 Gy fractions), which was felt to be a reasonable balance between anticipated local control and toxicity risk. We ceased treatment after 36 Gy due to acute mucositis, and the patient has been disease-free in the hard palate for at least 36 months. This suggests that PVL may be relatively radiosensitive, making this an attractive option for patients who do not want to undergo radical surgery. This also raises the possibility of dose de-escalation. Because one of the treatment goals was to avoid a highly morbid surgery, we would be cautious with dose de-escalation below what this patient received.

The patient did develop late ulceration requiring dexamethasone solution and topical lidocaine. Figure 6B shows the stark border of depapillation that appears to correspond with the posterior extent of the oral positioning stent. As seen in Figure 5B, there was less tongue-sparing posterior to the oral positioning stent. The surface of the dorsal tongue posterior to the positioning stent likely received about 48 Gy in 12 fractions, which corresponds to an EQD2 of 68 Gy using an alpha/beta ratio of three--likely accounting for the late toxicity. Practitioners could potentially avoid this late toxicity by crafting the positioning stent to extend posteriorly enough to cover the length of the hard palate. Care in fabrication and placement of the oral positioning stent is critical to reducing the likelihood of late toxicity given the high dose per fraction when treating with brachytherapy. 

## Conclusions

In conclusion, PVL is a progressive disease of the oral cavity that can be challenging to treat, with high recurrence rates after surgery and a high rate of malignant transformation. Brachytherapy with a custom surface mold is feasible; it may be an effective treatment for oral PVL; it also allows for optimal sparing of the uninvolved mucosa of the oral cavity. Collaboration with a prosthodontics specialist for fabrication of an oral positioning stent may lead to a more conformal therapy with better displacement of normal organs.
